# Persistent immune dysfunction during suppressive antiretroviral therapy: implications for analytical treatment interruption trials for chronic HIV infections in sub-Saharan Africa

**DOI:** 10.3389/fimmu.2026.1754996

**Published:** 2026-03-27

**Authors:** Patricia Kankundiye, Praiscillia Kia, Edward Kankaka, Rose Nabatanzi, Tokameh Mahmoudi, Damalie Nakanjako

**Affiliations:** 1 Department of Immunology and Molecular Biology, School of Biomedical Sciences, Makerere University College of Health Sciences, Kampala, Uganda; 2 Infectious Diseases Institute, Makerere University College of Health Sciences, Kampala, Uganda; 3 Africa-Europe Cluster of Excellence in Translational Science in Infection, Immunity and Inflammation (CoRE Triii), Makerere University College of Health Sciences, Kampala, Uganda; 4 Research Department, Rakai Health Sciences Project, Kalisizo, Uganda; 5 Department of Pathology, Erasmus Medical Centre, Rotterdam, Netherlands; 6 Department of Urology, Erasmus Medical Centre, Rotterdam, Netherlands; 7 Department of Medicine, School of Medicine, Makerere University College of Health Sciences, Kampala, Uganda

**Keywords:** broadly neutralizing antibodies (bnAbs), chronic HIV infections, HIV cure, HIV immunotherapy, HIV treatment interruption, persistent HIV-associated immune dysfunction, sub-Saharan Africa

## Abstract

**Background:**

Despite the success of long-term antiretroviral therapy (ART), immune dysfunction—including incomplete T helper cell recovery, immune activation, and inflammation—persists and may affect the benefits of analytical ART treatment interruption (ATI) trials among people living with human immunodeficiency virus (HIV) (PLHIV) in sub-Saharan Africa (SSA). This narrative review summarizes evidence of incomplete immune function recovery among PLHIV on long-term ART and potential interventions to enhance their ability to participate in ATI trials with immunotherapies in the quest for an HIV cure in SSA.

**Methods:**

A PubMed search query was used “([Immunity, Innate{MeSH Terms}] OR [Adaptive Immunity{MeSH Terms}] AND [HIV{MeSH Terms}] AND [Anti-HIV Agents{MeSH Terms}] AND [function{Title/Abstract} OR dysfunction{Title/Abstract} OR recovery{Title/Abstract} OR restoration{Title/Abstract} OR reconstitution{Title/Abstract} OR regeneration{Title/Abstract}])”, which retrieved 165 articles. These articles were filtered using an English-language filter, resulting in 160 papers. This query was translated to Web of Science and Google Scholar. In addition, we conducted a specific literature search on documented “cured” HIV cases globally to understand innate and adaptive immune functions relevant to supporting post-ART immunological viral control.

**Results:**

Persistent dysfunction of host innate and adaptive immunity during suppressive ART is reported in SSA HIV treatment cohorts. Natural killer (NK) cells, dendritic cells, and monocyte dysfunction potentially limit post-ART viral control. In addition, persistently impaired CD4 and CD8 T-cell proliferation capacity, immune activation, and exhaustion during ART may limit host HIV-specific responses during ATI trials with broadly neutralizing antibodies (bNAbs) and therapeutic vaccines, thereby increasing the risk of post-ART viral rebound. Similarly, adjunct therapies with TLR7 agonists, such as vesatolimod, could potentially increase cytotoxic capabilities of dendritic and natural killer cells to improve HIV latency reversal and viral clearance during ART.

**Conclusion:**

Innovative combination immunotherapies to avert persistent immune dysfunction, such as therapeutic vaccines, combination bNabs, enhancer of zeste homolog 2 (EZH2) inhibitors to boost CD8^+^ T-cell function and augment post-ART viral control, and latency reversal agents to increase cytotoxicity potential of dendritic cell and natural killer cells, among others, could potentially optimize HIV-specific CD8 T-cell cytotoxicity and viral clearance, and delay viral rebound during ATI trials in SSA.

## Introduction

People living with human immunodeficiency virus (HIV) (PLHIV) and combination antiretroviral therapy (ART) now have improved life expectancy (1, 2), and HIV has transitioned from a deadly virus into a chronic illness. The decrease in plasma HIV RNA levels, which permits partial restoration of immunological function, especially through the recovery of CD4^+^ T-cell numbers, is one of the successes of ART. However, despite long-term ART and virological suppression, 10% to 40% of people do not fully restore their immune system function, and immunological reconstitution is frequently insufficient (3). Incomplete immune function recovery exhibited by persistent immune activation and lingering inflammation remains an outstanding challenge contributing to morbidity and mortality from both acquired immunodeficiency syndrome (AIDS)-related and noncommunicable disease (NCD) comorbidity among PLHIV. Hence, there is a need to understand how persistent dysfunction of the innate and adaptive immune compartments may affect post-ART viral control during HIV cure trials, particularly in sub-Saharan Africa (SSA), where the majority of PLHIV initiate ART after chronic HIV infection.

Recent advances in immunotherapy, gene editing, and host-directed therapies have raised the likelihood of an HIV cure. A number of these approaches, including hematopoietic stem cell transplantation, therapeutic vaccines, latency-reversing drugs, and broadly neutralizing antibodies (bnAbs) to reduce or eliminate the viral reservoir, improve host immunity, and produce long-lasting viral remission following ART discontinuation, are under investigation. Despite these advancements, outcomes are still heterogeneous, with many therapies not providing reliable long-term HIV viral control after treatment interruption. Moreover, many of the analytical antiretroviral treatment interruption (ATI) trials are not directly transferable to the majority of PLHIV in SSA, where most individuals initiate ART during chronic HIV infection and where HIV infections are predominantly heterogeneous non-B subtypes and recombinants. The application of HIV cure strategies such as bNAbs, therapeutic vaccinations, and gene therapies is not yet well understood for chronic HIV infection in this region. Therefore, this narrative review analyzes persistent HIV-associated immune dysfunction during long-term ART and its implications for scaling up HIV cure interventions and immune therapies for chronic HIV infections in SSA, which constitutes over two-thirds of the world’s people living with HIV.

## Materials and methods

We did literature search in PubMed using the search query “([Immunity, Innate{MeSH Terms}] OR [Adaptive Immunity{MeSH Terms}] AND [HIV{MeSH Terms}] AND [Anti-HIV Agents{MeSH Terms}] AND [function{Title/Abstract} OR dysfunction{Title/Abstract} OR recovery{Title/Abstract} OR restoration{Title/Abstract} OR reconstitution{Title/Abstract} OR regeneration{Title/Abstract}])”. The search retrieved 165 articles, which were filtered using an English-language filter. Translation of this query to Web of Science and Google Scholar returned 160 papers. In addition, we conducted a specific literature search on the documented “cured” HIV cases globally to understand the innate and adaptive immune functions relevant to support post-ART immunological viral control.

## Results

### Persistent innate immune dysfunction during ART

Reconstitution of the innate immune system under long-term ART is generally insufficient, resulting in persistent alterations in natural killer cells (NK), monocytes, phagocytes, and mucosal innate lymphoid cells, as illustrated in **Table 1**. Studies indicate a trend among groups toward dysfunctional or hyperactivated NK subsets with impaired cytotoxicity capabilities, abnormal monocyte phenotypes, impaired phagocytic function, and a reduction of gut-resident innate populations (5, 9, 10). In addition, ongoing viral replication reduces the fraction of cytotoxic CD56dimCD16^+^ NK cells and promotes the emergence of dysfunctional NK cell responses (10, 13, 14), as outlined in **Table 1**. These alterations perpetuate systemic inflammation without facilitating the elimination of HIV-infected cells. These inherent deficiencies during long-term ART could potentially impede antibody- and Fc-mediated effector functions and increase the risk of rapid viral rebound following the cessation of treatment. Concerns may be heightened in sub-Saharan African contexts because of delayed ART initiation and HIV viral heterogeneity with several non-B subtype strains that predominate, amid other endemic coinfections including malaria, tuberculosis, and helminths (15, 16).

**Table 1:** Persistent Innate immune Dysfunction during treatment of chronic HIV infections.

Citation	Cohort Description	Dysfunction	Impact on Post-ART Control
Qian, F et al. (4)	29 Chronic HIV patients in China after 2 years of ART (15 with CD4<200 cells/μl and 14 with CD4>200 cells/μl	Increased killing ability of CD56^dim^ NK and significantly correlated with apoptosis of T lymphocytes	High apoptosis may deplete/exhaust T cells, reducing coordinated responses to reactivated virus; and risk shorter time-to-rebound
Nabatanzi, R. et al. (5)	HIV treatment cohort in Uganda; At least 12 years of ART	Increased ILC3 and decreased ILC1s and ILC2; Depletion of ILC High IL-1β, IL-6, IL-8, IL-18	Prolonged inflammation may reduce chances of durable remission after ATI
Luo, Z. et al. 2017 (6)	36 patients with chronic HIV in South Carolina after 14 years of ART; Nadir CD4 < 350 cells/µl	Increased NK cell activation of CD56dimCD16+ NK cells NK cells activation inversely correlated with the peripheral CD4+ counts	Hyperactivated NK cells with inefficient target clearance + low CD4 milieu predict fragile post-ART control; inflammation may accelerate rebound
Giuliani, E. et al. (7)	28 with chronic HIV infection of Caucasian origin; 2 years of ART (12 with CD4<350cells/μl and 16 with CD4 >500cells/μl	Increased CD56^bright^ NK cells Reduced Cytokine-induced IFN-γ production and NKp44 upregulation; T reg counts inversely correlated with autoreactive CD56^bright^ cells	Insufficient ADCC may favor rebound; Treg skew may further dampen effective clearance
Chen, P. et al. 2017 (8)	173 men including 63 chronic infected patients with ART Beijing, China The mean CD4/CD8 ratio was 1.07 of the chronic HIV patients on ART	Lower intermediate CD14++CD16+ monocyte subsets, the HLA-DR density and CD163 density on CD14++CD16+ monocytes	Disrupted monocyte function may hamper clearance of reactivated infected cells and promote viral rebound.
Frias, M. et al. (9)	39 (31 Male, 8 female) Chronic HIV patients in Spain; median CD4 count 258 cells/µl Three (3) years of ART	Incomplete Recovery of NK cell subsets with fewer potent cytotoxic NKs: Low percentage of total CD56^+^ and CD56^dim^ ; Low % of NK cells expressing NKp30 and NKp46	Poor activating-receptor signaling may cause weaker clearance of reactivated cells upon antiretroviral treatment interruption (ATI)
Bayigga, L. et al. (10)	211 (68% female) Ugandan cohort; after 4 years of ART; Median Nadir CD4 count<200/µl	Higher proportion of pro-inflammatory CD56^++^CD16^--^ NK cells; Low CD56^neg^ NK cells Might lead to bystander inflammation without effective killing to limit effective remission	Potential role for single-chain diabodies (scDbs) that target the HIV-1 envelope protein (Env) and the human type III Fcγ receptor (CD16 to promote NK **cell-**mediated elimination of HIV-1 reservoir *(Board et al 2024)*
Kløverpris, H. N. et al. 2014 (11)	147 patients including 83 chronic HIV infected in Durban, South Africa; ART duration not reported	Depletion ILC1 and ILC3 populations during chronic infection. Loss of ILC1 /ILC3 and gut-associated innate cells lowers the mucosal barrier	These may assist the virus remain in the body and may contribute to faster viral rebound
Michailidis, C. et al. 2012 (12)	100 patients in G. Gemimatas Athens General Hospital (60 (31 M/ 29 F), ART period not reported	Low phagocytic function and of oxidative burst of both monocytes and neutrophils; after ATI ,	Diminished first-line infection control and cell clearance, reducing residual viral containment

Moreover, host innate immunity remains relevant to improve the efficacy of HIV-1 cure strategies, with a particular focus on dendritic cells (DCs) and natural killer (NK) cells, including novel latency-reversing agents targeting DCs as well as DC-based strategies to enhance the clearance of HIV-infected cells by CD8^+^ T cells and other strategies to improve the killing activity of NK cells (17). For example, two single-chain diabodies (scDbs) that target the HIV-1 envelope protein (Env) and the human type III Fcγ receptor (CD16) were shown to promote NK cell**-**mediated elimination of HIV-1 reservoir cells that was dependent on latency reversal, through robust HIV-1-specific NK cell activation and NK cell-mediated lysis of HIV-infected cells (18). These bispecific antibodies mediated the elimination of up to 72% of intact provirus^+^ CD4^+^ T cells from people receiving suppressive ART *ex vivo*, thus representing promising new therapeutics for HIV-1 reservoir reduction strategies. In addition, the evaluation of 3BNC117-Db in HIV-1-infected humanized mice suggested that bispecific antibodies may also have potential application for limiting reservoir establishment during acute HIV-1 infection or reducing reservoir size during chronic HIV-1 infection (18). Therefore, HIV-1-specific scDbs merit further evaluation as potential therapeutics for the clearance of the latent reservoir in SSA HIV treatment cohorts (**Table 1**). Protracted exposure with repeated antigenic stimulation by the endemic infections of malaria, tuberculosis, and intestinal helminths, together with delayed ART initiation, synergizes to fuel activation of the innate immune system. These exposures exacerbate microbial translocation and monocyte/macrophage activation, perpetuating elevated biomarkers long after ART-mediated viral suppression. Persistently dysregulated innate immunity contributes to systemic inflammation, non-AIDS comorbidities (e.g., cardiovascular disease and neurocognitive impairment), and may influence the kinetics of viral rebound during ATI. The continued elevation of innate biomarkers after ART highlights the need to integrate these metrics into monitoring frameworks and to explore targeted interventions that modulate innate activation prior to and during cure-directed trials (5).

### Persistent adaptive immune dysfunction during ART

Individuals who initiated ART after advanced chronic HIV infection (with low nadir CD4 levels) continue to exhibit dysfunction of the adaptive immune system despite suppressive ART, as illustrated in **Table 2**. The primary dysfunctions include reduced naïve and recent thymic emigrant CD4 T-cell pools, persistent T-cell activation, accumulation of exhausted and senescent CD4 and CD8 T cells, increased regulatory T-cell populations, and diminished diversity in B-cell receptors (19, 24). If left unaddressed, these persistent dysfunctions are likely to limit the scope and effectiveness of HIV-specific responses required for post-ART viral control following treatment interruption.

**Table 2:** Persistent Adaptive Immune dysfunction during ART for chronic HIV infections.

Citation	Cohort	Description of Dysfunction	Implications for Post ART Viral control
Qian, F. et al. (4)	29 Chronic HIV patients from China (15 had CD4 counts<200 cells/ µl and 14 had> 200 cells / µl); 2 years of ART	CD4+ and CD8+ Cell-surface expression of programmed death-1 (PD-1) was markedly elevated in CD4; particularly with CD4 counts< 200 cells / µl	T-cell exhaustion limits proliferation/killing of reactivated cells; and likely predisposes to earlier rebound
Xiao, Q. et al. (19)	92 patients with Chronic HIV infection in China with CD4 counts <500 cells / µl	Low CD4+ naive T cells and Recent Thymic Emmigrant CD4 + T cells	Weaker antibody maturation and CD8 support; likely less durable control after antiretroviral treatment interuption (ATI)
Jia, J. et al. 2022 (20)	2 years of ART (10 with >500 cells/μl, 8 with <350 cells/μl) and 8 male Healthy controls; in China	Decreased B Cell Receptor diversity	Potentially limited spectrum of antibody responses, complicating the management of diverse or escape variants post ATI; May also affect effectiveness of therapeutic vaccinations or bNAbs
Jimenez, V. et al. 2016	95 patients with Chronic HIV from Amsterdam	High Tregs, CD4+ PD-1 expression and CD4+CD38+ HLA-DR +	T-cell activation and exhaustion may limit efficient HIV-specific responses and promote faster Post ART viral rebound
Nakanjako, D. et al. 2013 (21)	211 patients undetectable VL after 4 years of ART	Low T-cell proliferation determined by the extent of carboxyfluorescein diacetate succinimidyl ester (CFSE) dye	Robust CD8 proliferation has associated more durable post-ART viral control
Nakanjako, D. et al. 2011 (22)	128 patients with Chronic HIV in Ugandan after 4 years of ART	Persistent high CD4+ T cell and CD8 T cell activation	Prolonged inflammation may impair effective HIV-specific cytotoxic function
Crawley, A.M., et al. 2012 (23)	35 Chronic HIV patients at least 1 year of ART in Rome, Italy	Reduction of Thymic naïve CD4 T cells	Low-quality responses to viral antigens; including diminished vaccine/bnAb synergy

Chronic HIV exposure leads to sustained upregulation of inhibitory receptors (including Programmed Cell Death Protein 1 (PD-1), CTLA-4, LAG-3) on CD4⁺ and CD8⁺ T cells and persistent coexpression of immune activation markers (Human Leukocyte Antigen) sub-region D related (HLA-DR) and Cluster of Differentiation 38 (CD38)). This phenotype reflects a state of exhaustion characterized by reduced proliferative capacity, decreased cytokine production, and compromised effector functions. Elevated levels of systemic inflammation also impede adaptive responses. Persistent IL-6, CRP, MCP-1, and neopterin are associated with ongoing immune activation and impaired memory T-cell functionality even after years of suppression. These cytokines drive continued T-cell activation and turnover, fostering exhaustion and impairing long-term immunologic memory (15).

Chronic inflammation affects antigen-specific T-cell responses and vaccine immunity. ART-treated individuals show altered transcriptional profiles and function within memory CD4⁺ T cells, yielding reduced cytokine responses to recall antigens and vaccines despite ART-mediated viral suppression. This qualitative impairment extends beyond HIV-specific responses and has implications for therapeutic vaccination and other immune-based cure strategies (16). Moreover, the interplay between HIV-TB and other endemic pathogens can lead to paradoxical immune activation that is simultaneously ineffective at pathogen control (17).

Adaptive immune dysfunction undermines the efficacy of therapeutic vaccines, immune checkpoint modulation, and bNAb HIV cure strategies by limiting the development of effective cytotoxic T-cell responses and high-affinity antibody repertoires. These data suggest that many ART-treated patients in SSA would need agents to boost their HIV-specific CD8 T-cell responses to sustain HIV viral control after treatment interruption in the quest for an HIV cure. A need therefore exists for more controlled monitoring of immune responses and stringent viral rebound measurements to understand the benefits of ATI trials among HIV treatment cohorts in SSA. For example, integrating assessment of biomarkers of adaptive immune dysfunction, such as immune activation and T-cell exhaustion markers, as well as HIV-specific CD8 proliferation and cytotoxicity, would be essential for SSA populations with unique immunologic landscapes (15).

### Immune therapies for ART-treated individuals with chronic HIV infection

**Table 3** presents key immunologic curative options currently being evaluated, which encompass broadly neutralizing antibodies (bNAbs), therapeutic vaccinations, innate immune agonists, cytokine-based therapies, as well as gene- and cell-based strategies. Monotherapy with individual bNAbs or latency reversal agents (LRAs) has generally produced only transient effects. In contrast, combination bNAb regimens and multifaceted strategies that include therapeutic vaccines or TLR7 agonists have demonstrated more substantial delays in viral rebound among certain early-treated, primarily subtype B cohorts (17, 18). We also highlight the need to monitor specific biomarkers of HIV-specific T-cell function, such as CD8 T-cell proliferation using CFSE dye and intracellular expression of Ki-67 (25), IFN-γ Gag/conserved elements (CE)-specific CD4^+^/CD8^+^ T-cell responses and upregulated Gag/CE-specific immune activation (HLA-DR and CD38) and exhaustion markers (PD-1, CD39), which could be used as predictors of post-ART viral control during ATI trials with combination bNAbs and therapeutic vaccines (25, 26, 33). In addition, expression of molecular signatures of superior CD8^+^ T-cell function (genes and surface proteins associated with stemness, CD45RA, CD62L, CCR7, CD27, TCF7) could potentially be used as predictors of post-ART viral control (25).

**Table 3:** Potential HIV immunotherapies: antiretroviral treatment interruption (ATI) trials in sub-Saharan Africa.

Citation	Cohort description	Immunotherapy	Implication for PLWHIV in SSA
Kiani Z et al. (25)	12 participants from four ATI trials in the USA	bNABs (3BNC117 and/or10-1074	CD8 T-cell functionality is critical for post-ART viral control
	Seven achieved postintervention viral control (PIC) “controllers”	Stronger CD8^+^ T-cell proliferative capacity (low CFSE dye) in controllers relative to noncontrollers	Individuals with persistent CD8 T-cell dysfunction are unlikely to control the virus even with combination bNAb interventions
	Five did not achieve PIC “noncontrollers”	HIV epitope-specific CD8^+^ T cells in controllers had higher CD45RA^+^CD62L^+^ stem-like memory cells than noncontrollers	
		Higher expression of molecular signatures of superior CD8^+^ T-cell function in controllers (genes and surface proteins associated with stemness CD45RA, CD62L, CCR7, CD27, TCF7)	
Peluso MJ et al. (26)	10 individuals in the USA (three started ART within 30 days of HIV infection, four within 1–6 months of infection, and three after 6 months of infection), undetectable VL, CD4 > 500; median length of ATI 36.7 weeks (range: 14.7–77.1)	Interventions:	Therapeutic vaccines elicited new or boosted pre-existing IFN-γ Gag/CE-specific CD4^+^ and CD8^+^ responses and upregulated Gag/CE-specific activation markers (PD-1, CD39)
		a) Therapeutic vaccine with HIV-Gag conserved element to enhance HIV-specific T-cell responses	
		b) Combination of two long-acting bNAbs (10.1074 and VRC07-523LS) and a potential latency reversal agent (lefitolimod)	
			Robust HIV-specific CD4^+^ and CD8 responses are critical for post-ART viral control
		Seven out of 10 exhibited post-ART control; high bNAb susceptibility and exposure were associated with delayed rebound Three controllers had no measurable reservoir	
Gramatica A et al. (27)	Inhibition of enhancer of zeste homolog 2 (EZH2) with tazemetostat	Tazemetostat promotes sustained skewing of CD8^+^ T cells toward less-differentiated and exhausted phenotypes	Combination interventions with tazemetostat could potentially boost CD8^+^ T-cell function and augment post-ART viral control
Bailon L et al. (28)	Clinical trial; 50 men with chronic HIV infection were enrolled in Spain; the median CD4 T-cell count was 882 cells/µL. ART duration was 43 months for the intervention group and 41 months for the placebo group; reservoir monitoring was performed	HTI-based therapeutic vaccine (ChAdOx1.HTI plus MVA.HTI.CCMM) combined with a Toll-like receptor 7 (TLR7) agonist. HIV-1 rebound was detected in all participants after ART discontinuation	Therapeutic vaccines may be immunogenic but not sufficient alone, especially in SSA, where individuals initiate ART at low CD4 counts
		Two participants were lost to follow-up	
Rosás-Umbert M et al. (29)	ART initiated with or without adjunct bNAb 3BNC117 treatment	Frequencies of Pol- and Gag-specific CD8^+^ T cells and Gag-induced interferon-γ responses were higher in individuals who received adjunctive 3BNC117 compared with ART-alone at 3 and 12 months after starting ART	Increased HIV-1-specific immunity is associated with partial or complete ART-free virologic control during treatment interruption for up to 4 years
Gaebler C et al. (30)	Clinical trial; 26 patients from the USA were enrolled; reservoir monitoring was performed; median duration on ART was 10 years (range: 1–29); 88% men; mean nadir CD count was 359 cells/µL; CD4 count at ATI was 729 and 660 cells/µL at rebound	bNAbs (3BNC117 and 10–1,074) were used; some patients had viral suppression after ATI for 7 weeks, 28 weeks, and others for the whole 48 weeks of the study One participant was lost to follow-up	Combination bNAb therapy is better for functional remission
Sengupta D et al. (31)	31 adults in the USA with chronic HIV infection were enrolled; ART was administered for 2.7 years; reservoir monitoring was performed	Latency reversal agents: TLR7 agonist vesatolimod; median time to viral rebound was 4.3 weeks for a threshold of ≥ 50 copies RNA/mL and 5.1 weeks for a threshold of ≥ 200 copies RNA/mL	A modest delay in viral rebound is best when used in combination with other strategies
Sengupta D et al. (32)	Phase 1b randomized, double-blind, placebo-controlled clinical trial of an oral TLR7 agonist, vesatolimod, in HIV-1-infected controllers on ART (*n* = 17)	Vesatolimod was associated with the induction of immune cell activation, decreases in intact proviral DNA during ART, and a modest increase in time to rebound after ART was interrupted	Increased dendritic cell and natural killer cell cross-talk and an increase in cytotoxicity potential after vesatolimod dosing
Scheid JF et al. (8)	52 patients with chronic HIV in the USA were enrolled; nadir CD4 count was > 200 cells/μL; ART duration was ≥ 12 months. Mean CD4 at ATI start was 747 cells/µL, and mean CD4 change to rebound was − 127 cells	bNAb 3BNC117 was used; 19 weeks of remission were achieved after four infusions of the antibody; rebound was due to resistance to the antibody	Combination instead of monotherapy delays viral rebound after ART interruption

## Discussion

### Persistent immune dysfunction during ART: what are the implications for ART treatment trials in SSA?

Despite several breakthroughs in treatment and management, HIV continues to be a major global health concern. The number of AIDS-related fatalities has decreased from 1.8 million in 2000 to 630,000, and new HIV infections from 2.8 to 1.3 million (34). However, persistent immune dysfunction demonstrated in settings of suppressive ART, including NK and DC dysfunction, monocyte activation, and inflammasome induction, is associated with ongoing CD4 decline, limited HIV-specific cytotoxicity, and increased risk of early viral rebound after ART discontinuation (17, 18, 24, 35, 36). Monocytes/macrophages and dendritic cells play a role in the pathological alterations affecting the quality of CD4^+^ T cells (24). Continuous inflammation and activation of monocytes and other innate immune cells are likely linked to prolonged T-cell exhaustion and weakened effector functions despite long-term suppressive ART (154). Similarly, a greater percentage of proinflammatory CD56brightCD16dim NK cells has been demonstrated among HIV-infected individuals receiving suppressive ART with suboptimal immune response relative to immune responders (10), indicating a potential increase of autoreactive CD56bright NK cells associated with diminished Treg-mediated control (7) and increased T-cell apoptosis associated with high cytotoxicity of CD56dim NK cells (4). These findings suggest that if left unmanaged, changes in NK cell phenotypes and function, including proinflammatory or autoreactive phenotypes and increased cytotoxicity, could have negative effects on post-ART HIV viral control after HIV treatment interruption (10, 24, 37, 38).

Noteworthy is the fact that agents that reduce HIV-associated immune activation and exhaustion may be beneficial as adjunct therapies to ART to improve recovery of host T-cell function required for effective, robust viral clearance during ATIs with combination bNAbs and therapeutic vaccines (25, 26). In a randomized crossover placebo-controlled trial in Uganda, we demonstrated that high-dose atorvastatin among ART-treated adults reduced immune activation (coexpression of HLA-DR and CD38) and exhaustion (PD-1 expression) relative to placebo (39). Moreover, there is evidence that daily pitavastatin calcium may prevent the occurrence of severe cardiovascular disease (including myocardial infarction, hospitalization for unstable angina, stroke, transient ischemic attack, peripheral arterial ischemia, and revascularization) among ART-treated adults in the USA (40, 41). Hence, there is a need for well-characterized studies to better understand the role of anti-immune activation agents, such as stains, on HIV-specific T-cell functions relevant for post-ART viral control. In addition, evidence is needed regarding the reduction of immune activation and the incidence of non-AIDS comorbidities and NCDs among adults aging with HIV in SSA.

### Implications for novel immunotherapies for ART-treated individuals in SSA

Of note, a number of HIV cure remission cases have documented evidence of either a sterilizing cure or long-term functional remission in high-income countries. For example, the Berlin, City of Hope National Medical Center, and Düsseldorf patients who show different biological and clinical pathways to long-lasting remission. These cases provide unique insight into the circumstances under which a cure may be possible, such as detection of HIV infection within hours or days, immediate initiation of ART, ART regimen, and particularly for subtype B infections (42). Many of these settings are not directly transferable to the HIV epidemic in SSA, which is predominated by chronic HIV infections, initiation of ART at advanced stages of HIV disease, and heterogeneity of non-B HIV subtypes. Nevertheless, the cases provide important knowledge about viral immune pathogenesis that can be utilized to translate these rare successes into widely applicable solutions for both acute and chronic HIV infections globally.

Our findings suggest that in high-HIV-burden, resource-limited regions predominantly featuring non-B subtypes, such as SSA, future cure initiatives should prioritize potentially scalable ATI trials with combination of successful interventions, including long-acting bNAb combinations, transportable vaccination platforms, and oral innate immunity agonists, contingent upon their integration with strategies addressing underlying immune dysfunction and their alignment with local viral diversity and health system capacity. In addition, the design of ATI trials in SSA should include careful monitoring of chronic inflammation and immune activation markers (including IP-10, sCD14, LPS, and caspases) that may be affected by the endemic coinfections that include tuberculosis, malaria, and intestinal helminths. In addition, HIV treatment cohorts in SSA that are predominantly young women provide opportunities to understand age and sex differences in host immune capabilities for post-ART viral control and predictors of early viral rebound.

### Limitations

We were limited by the critical gap in host and viral immunologic data in SSA and nonsubtype B acute and chronic HIV infections, particularly concerning advanced immunotherapies such as bNAbs, therapeutic vaccines, and gene and stem cell treatments, among others. The molecular evidence supporting immune dysfunction and potential curative options is primarily derived from homogeneous, small cohorts in high-income countries, with specific clinical characteristics, early treatment cohorts, or transplant recipients, which are not directly transferable to HIV epidemic settings in SSA. Similarly, gene editing, chimeric antigen receptor T (CAR-T) cell therapy (43, 44), and CCR5Δ32-based transplantation provide compelling mechanistic proof-of-concept (45), although they are challenging, expensive, and limited to specialized environments that may not be easily accessible in many low- and middle-income countries. This is consistent with a recent review that emphasized limitations in training, infrastructure, and funding for HIV cure research in Africa (46). It is therefore critical to advocate (to patients, health workers, and funders) to expand inclusion of SSA HIV treatment cohorts in HIV cure trials to generate the much-needed immunological data in SSA, where host immune profiles and viral diversity are different from those in high-income countries. Generating more immunological data from SSA cohorts would greatly inform the translation of some of the lessons and benefits of ATI trials in high-income countries to resource-limited settings that are still facing a high burden of PLHIV.

## Conclusion

Innovative combination immunotherapies to avert persistent immune dysfunction, such as therapeutic vaccines, combination bNabs, enhancer of zeste homolog 2 (EZH2) inhibitors like tazemetostat to boost CD8^+^ T-cell function and augment post-ART viral control, and latency reversal agents like vesatolimod to increase cytotoxicity potential of dendritic and natural killer cells, among others, could potentially optimize HIV-specific CD8 T-cell cytotoxicity and viral clearance, and delay viral rebound during ATI trials in SSA.

## References

[1] Katz IT Maughan-Brown B . Improved life expectancy of people living with HIV: who is left behind? Lancet HIV. (2017) 4:e324-e6. doi: 10.1016/S2352-3018(17)30086-3, PMID: 28501496 PMC5828160

[2] Wandeler G Johnson LF Egger M . Trends in life expectancy of HIV-positive adults on antiretroviral therapy across the globe: comparisons with general population. Curr Opin HIV AIDS. (2016) 11:492–500. doi: 10.1097/COH.0000000000000298, PMID: 27254748 PMC5055447

[3] Zhang W Ruan L . Recent advances in poor HIV immune reconstitution: what will the future look like? Front Microbiol. (2023) 14:1236460. doi: 10.3389/fmicb.2023.1236460, PMID: 37608956 PMC10440441

[4] Qian F Hu S Zhu Y Wang Y Liu J Qiao J . CD56(dim) NK cell is an important factor in T cell depletion of cART-treated AIDS patients. Int J Gen Med. (2022) 15:4575–83. doi: 10.2147/IJGM.S356771, PMID: 35535146 PMC9078362

[5] Nabatanzi R Cose S Joloba M Jones SR Nakanjako D . Effects of HIV infection and ART on phenotype and function of circulating monocytes, natural killer, and innate lymphoid cells. AIDS Res Ther. (2018) 15:7. doi: 10.1186/s12981-018-0194-y 29544508 PMC5853105

[6] Luo Z Li Z Martin L Hu Z Wu H Wan Z . Increased natural killer cell activation in HIV-infected immunologic non-responders correlates with CD4+ T cell recovery after antiretroviral therapy and viral suppression. PLoS One. (2017) 12:e0167640. doi: 10.1371/journal.pone.0167640, PMID: 28076376 PMC5226712

[7] Giuliani E Vassena L Di Cesare S Malagnino V Desimio MG Andreoni M . NK cells of HIV-1-infected patients with poor CD4(+) T-cell reconstitution despite suppressive HAART show reduced IFN-γ production and high frequency of autoreactive CD56(bright) cells. Immunol Lett. (2017) 190:185–93. doi: 10.1016/j.imlet.2017.08.014, PMID: 28826739

[8] Chen P Su B Zhang T Zhu X Xia W Fu Y . Perturbations of Monocyte Subsets and Their Association with T Helper Cell Differentiation in Acute and Chronic HIV-1-Infected Patients. Front Immunol. (2017) 8. doi: 10.3389/fimmu.2017.00272, PMID: 28348563 PMC5347116

[9] Frias M Rivero-Juarez A Gordon A Camacho A Cantisan S Cuenca-Lopez F . Persistence of pathological distribution of NK cells in HIV-infected patients with prolonged use of HAART and a sustained immune response. PLoS One. (2015) 10:e0121019. doi: 10.1371/journal.pone.0121019, PMID: 25811634 PMC4374841

[10] Bayigga L Nabatanzi R Sekiziyivu PN Mayanja-Kizza H Kamya MR Kambugu A . High CD56++CD16- natural killer (NK) cells among suboptimal immune responders after four years of suppressive antiretroviral therapy in an African adult HIV treatment cohort. BMC Immunol. (2014) 15:2. doi: 10.1186/1471-2172-15-2, PMID: 24483185 PMC3915033

[11] Kløverpris HN McGregor R McLaren JE Ladell K Stryhn A Koofhethile C . Programmed death-1 expression on HIV-1-specific CD8+ T cells is shaped by epitope specificity, T-cell receptor clonotype usage and antigen load. AIDS. (2014) 28:2007–21. doi: 10.1097/QAD.0000000000000362, PMID: 24906112 PMC4166042

[12] Michailidis C Giannopoulos G Vigklis V Armenis K Tsakris A Gargalianos P . Impaired phagocytosis among patients infected by the human immunodeficiency virus: implication for a role of highly active anti-retroviral therapy. Clin Exp Immunol. (2012) 167:499–504. doi: 10.1111/j.1365-2249.2011.04526.x, PMID: 22288593 PMC3374282

[13] Mavilio D Benjamin J Daucher M Lombardo G Kottilil S Planta MA . Natural killer cells in HIV-1 infection: dichotomous effects of viremia on inhibitory and activating receptors and their functional correlates. Proc Natl Acad Sci U S A. (2003) 100:15011–6. doi: 10.1073/pnas.2336091100, PMID: 14645713 PMC299884

[14] Mavilio D Lombardo G Benjamin J Kim D Follman D Marcenaro E . Characterization of CD56-/CD16+ natural killer (NK) cells: a highly dysfunctional NK subset expanded in HIV-infected viremic individuals. Proc Natl Acad Sci U S A. (2005) 102:2886–91. doi: 10.1073/pnas.0409872102, PMID: 15699323 PMC549494

[15] Bhengu KN Naidoo P Singh R Mpaka-Mbatha MN Nembe N Duma Z . Immunological interactions between intestinal helminth infections and tuberculosis. Diagnostics. (2022) 12:2676. doi: 10.3390/diagnostics12112676, PMID: 36359526 PMC9689268

[16] Hartgers Fc Yazdanbakhsh M . Co-infection of helminths and malaria: modulation of the immune responses to malaria. Parasite Immunol. (2006) 28:497–506. doi: 10.1111/j.1365-3024.2006.00901.x, PMID: 16965285

[17] Board NL Moskovljevic M Wu F Siliciano RF Siliciano JD . Engaging innate immunity in HIV-1 cure strategies. Nat Rev Immunol. (2022) 22:499–512. doi: 10.1038/s41577-021-00649-1, PMID: 34824401

[18] Board NL Yuan Z Wu F Moskovljevic M Ravi M Sengupta S . Bispecific antibodies promote natural killer cell-mediated elimination of HIV-1 reservoir cells. Nat Immunol. (2024) 25:462–70. doi: 10.1038/s41590-023-01741-5, PMID: 38278966 PMC10907297

[19] Xiao Q Yu F Yan L Zhao H Zhang F . Alterations in circulating markers in HIV/AIDS patients with poor immune reconstitution: Novel insights from microbial translocation and innate immunity. Front Immunol. (2022) 13:1026070. doi: 10.3389/fimmu.2022.1026070, PMID: 36325329 PMC9618587

[20] Jia J Zhao Y Yang JQ Lu DF Zhang XL Mao JH . Naïve B cells with low differentiation improve the immune reconstitution of HIV-infected patients. iScience. (2022) 25:105559. doi: 10.1016/j.isci.2022.105559, PMID: 36465118 PMC9708917

[21] Nakanjako D Ssewanyana I Nabatanzi R Kiragga A Kamya MR Cao H . Impaired T-cell proliferation among HAART-treated adults with suboptimal CD4 recovery in an African cohort. BMC Immunol. (2013) 14:26. doi: 10.1186/1471-2172-14-26, PMID: 23786370 PMC3706234

[22] Nakanjako D Ssewanyana I Mayanja-Kizza H Kiragga A Colebunders R Manabe YC . High T-cell immune activation and immune exhaustion among individuals with suboptimal CD4 recovery after 4 years of antiretroviral therapy in an African cohort. BMC Infect Dis. (2011) 11:43. doi: 10.1186/1471-2334-11-43, PMID: 21299909 PMC3065409

[23] Crawley AM Angel JB . The influence of HIV on CD127 expression and its potential implications for IL-7 therapy. Semin Immunol. (2012) 24:231–40. doi: 10.1016/j.smim.2012.02.006, PMID: 22421574

[24] Yan L XK Xiao Q Tuo L Luo T Wang S Yang R . Cellular and molecular insights into incomplete immune recovery in HIV/AIDS patients. Front Immunol. (2023) 14. doi: 10.3389/fimmu.2023.1152951, PMID: 37205108 PMC10185893

[25] Kiani Z Urbach JM Wisner H Olatotse MJ Chang DY Acklin JA . CD8+ T cell stemness precedes post-intervention control of HIV viraemia. Nature. (2026) 650:196–204. doi: 10.1038/s41586-025-09932-w, PMID: 41326735 PMC12872466

[26] Peluso MJ Sandel DA Deitchman AN Kim SJ Dalhuisen T Tummala HP . Combination immunotherapy induces post-intervention control of HIV. Nature. (2026) 650:187–95. doi: 10.1038/s41586-025-09929-5 41326736 PMC12872443

[27] Gramatica A Miller IG Ward AR Khan F Kemmer TJ Weiler J . EZH2 inhibition mitigates HIV immune evasion, reduces reservoir formation, and promotes skewing of CD8+ T cells toward less-exhausted phenotypes. Cell Rep. (2025) 44:115652. doi: 10.1016/j.celrep.2025.115652 40333189 PMC13088201

[28] Bailón L Alarcón-Soto Y Arribas JR Curran A Llano A Cedeño S . Predictors of virological outcomes after analytical interruption of antiretroviral therapy and HTI vaccination in early treated people with HIV-1. Commun Med (Lond). (2025) 6:11. doi: 10.1038/s43856-025-01266-y 41361072 PMC12775531

[29] Rosás-Umbert M Gunst JD Pahus MH Olesen R Schleimann M Denton PW . Administration of broadly neutralizing anti-HIV-1 antibodies at ART initiation maintains long-term CD8+ T cell immunity. Nat Commun. (2022) 13:6473. doi: 10.1038/s41467-022-34171-2 36309514 PMC9617872

[30] Gaebler C Nogueira L Stoffel E Oliveira TY Breton G Millard KG . Prolonged viral suppression with anti-HIV-1 antibody therapy. Nature. (2022) 606:368–374. doi: 10.1038/s41586-022-04597-1 35418681 PMC9177424

[31] SenGupta D Brinson C DeJesus E Mills A Shalit P Guo S . The TLR7 agonist vesatolimod induced a modest delay in viral rebound in HIV controllers after cessation of antiretroviral therapy. Sci Transl Med. (2021) 13:eabg3071. doi: 10.1126/scitranslmed.abg3071 34162752

[32] Scheid JF Horwitz JA Bar-On Y Kreider EF Lu CL Lorenzi JC . HIV-1 antibody 3BNC117 suppresses viral rebound in humans during treatment interruption. Nature. (2016) 535:556–560. doi: 10.1038/nature18929 27338952 PMC5034582

[33] Peluso MJ Sandel DA Deitchman AN Kim SJ Dalhuisen T Tummala HP . Correlates of HIV-1 control after combination immunotherapy. Nature. (2026) 650:187–95. doi: 10.1038/s41586-025-09929-5, PMID: 41326736 PMC12872443

[34] UNAIDS . (2023). UNAIDS DATA 2023. Geneva: UNAIDS. Available online at: https://www.unaids.org/sites/default/files/media_asset/data-book-2023_en.pdf

[35] Cai CW Sereti I . Residual immune dysfunction under antiretroviral therapy. Semin Immunol. (2021) 51:1–22. doi: 10.1016/j.smim.2021.101471, PMID: 33674177 PMC8410879

[36] Lisco A Wong CS Lage SL Levy I Brophy J Lennox J . Identification of rare HIV-1-infected patients with extreme CD4+ T cell decline despite ART-mediated viral suppression. JCI Insight. (2019) 4:1–13. doi: 10.1172/jci.insight.127113, PMID: 30996137 PMC6538352

[37] Caligiuri MA . Human natural killer cells. Blood. (2008) 112:461–9. doi: 10.1182/blood-2007-09-077438, PMID: 18650461 PMC2481557

[38] Crinier A Narni-Mancinelli E Ugolini S Vivier E . SnapShot: natural killer cells. Cell. (2020) 180:1280–.e1. doi: 10.1016/j.cell.2020.02.029, PMID: 32200803

[39] Nakanjako D Ssinabulya I Nabatanzi R Bayigga L Kiragga A Joloba M . Atorvastatin reduces T-cell activation and exhaustion among HIV-infected cART-treated suboptimal immune responders in Uganda: a randomised crossover placebo-controlled trial. Trop Med Int Health. (2015) 20:380–90. doi: 10.1111/tmi.12442, PMID: 25441397 PMC4529480

[40] Grinspoon SK Fitch KV Zanni MV Fichtenbaum CJ Umbleja T Aberg JA . Pitavastatin to prevent cardiovascular disease in HIV infection. N Engl J Med. (2023) 389:687–99. doi: 10.1056/NEJMoa2304146, PMID: 37486775 PMC10564556

[41] Grinspoon SK Ribaudo HJ Douglas PS . Pitavastatin and cardiovascular disease in HIV. Reply. N Engl J Med. (2023) 389:e46. doi: 10.1056/NEJMc2400870, PMID: 37991868

[42] Lihana R Ssemwanga D Abimiku AL Ndembi N . Update on HIV-1 diversity in africa: A decade in review. AIDS Rev. (2012) 14:83–100. 22627605

[43] Campos-Gonzalez G Martinez-Picado J Velasco-Hernandez T Salgado M . Opportunities for CAR-T cell immunotherapy in HIV cure. Viruses. (2023) 15:1–13. doi: 10.3390/v15030789, PMID: 36992496 PMC10057306

[44] Mao Y Liao Q Zhu Y Bi M Zou J Zheng N . Efficacy and safety of novel multifunctional M10 CAR-T cells in HIV-1-infected patients: a phase I, multicenter, single-arm, open-label study. Cell Discov. (2024) 10:49. doi: 10.1038/s41421-024-00658-z, PMID: 38740803 PMC11091177

[45] Gupta RK Peppa D Hill AL Gálvez C Salgado M Pace M . Evidence for HIV-1 cure after CCR5Δ32/Δ32 allogeneic haemopoietic stem-cell transplantation 30 months post analytical treatment interruption: a case report. Lancet HIV. (2020) 7:e340-e7. doi: 10.1016/S2352-3018(20)30069-2, PMID: 32169158 PMC7606918

[46] Nakanjako D Kankaka EN Lungu C Galiwango RM Reynolds SJ Mahmoudi T . HIV cure research contributions from Africa in the last three decades. Front Immunol. (2025) 16:1576667. doi: 10.3389/fimmu.2025.1576667, PMID: 40861434 PMC12370771

